# Unveiling patenting strategies of therapeutics and vaccines: evergreening in the context of COVID-19 pandemic

**DOI:** 10.3389/fmed.2023.1287542

**Published:** 2023-12-06

**Authors:** María Lorena Bacigalupo, María Florencia Pignataro, Carolinne Thays Scopel, Sergiy Kondratyuk, Othoman Mellouk, Gabriela Costa Chaves

**Affiliations:** ^1^Independent Researcher, Rio Negro, Argentina; ^2^Independent Researcher, Buenos Aires, Argentina; ^3^Independent Researcher, Rio de Janeiro, Brazil; ^4^International Treatment Preparedness Coalition Global (ITPC-Global), Bryanston, South Africa; ^5^Independent Researcher, Ferney Voltaire, France

**Keywords:** Evergreening, COVID-19, health technologies, SARS-CoV-2, patenting strategies, monopoly, patent examination

## Abstract

In the pharmaceutical sector, evergreening is considered a range of practices applied to extend monopoly protection on existing products. Filing several patent applications related to the same active pharmaceutical ingredient (API) is one of the most common manifestations of evergreening. During the COVID-19 pandemic, several health technologies were developed. This study aimed to analyze the extension of evergreening for selected health technologies for SARS-CoV-2 through patent filing strategies. Starting with the selection of three antivirals, one biological and two vaccines, a patent landscape was built based on public and private databases. Regarding these selected technologies, we analyzed some of the evergreening strategies used by different applicants, academic institutions or pharmaceutical companies and found a total of 29 applications (10 after the pandemic) for antivirals, 3 applications for a biological drug (1 after the pandemic), and 41 applications for vaccines (23 after the pandemic). Despite differences among the technologies, a common aspect found in all analyzed cases is the intense patent filing after the pandemic, aligned to the fact that those technologies were moving through the R&D process up to regulatory approval. The evergreening approach pursued has already been found in other diseases, with the risk of monopoly extension and also bringing legal uncertainty due to the lack of transparency of newer patent applications covering specific medical indications. Therefore, efforts to address evergreening should be pursued by countries, including the adoption of a public health approach to the patent examination of those technologies to prevent the granting of undeserved patents.

## 1 Introduction

Since December 2019, the world has witnessed the beginning of the severe acute respiratory syndrome coronavirus 2 (SARS-CoV-2) pandemic, with 769.4 million accumulated confirmed cases and 6.5 million deaths by August 2023 (1). In such a global public health crisis, there was an unprecedented race for the development of health tools to change the course of the crisis (2).

Despite the emergency authorization for some vaccines by the end of 2020, inequity in access between high-income (HIC) and low- and middle-income countries (LMIC) to COVID-19 health technologies has been the mark of this pandemic and the consequences of the monopoly situation on life-saving tools (3). This was reflected in the global scarcity of manufacturing those technologies, including diagnostics and therapeutics, and in the lack of a public health approach to the supply (4).

The development of COVID-19 technologies builds from decades of research & development (R&D) with key contributions from public funding (5–7). For example, almost US$ 1 billion of US taxpayers was invested in the Moderna vaccine R&D process (7). However, those investments were not followed with a commitment to ensure equitable global access (8). The inequitable distribution of COVID-19 vaccines was considered by the World Health Organization (WHO) “a moral and global security failure with health and economic consequences” (9).

Although initially the indication was that the approach would not be “business as usual” during the pandemic, quickly this proved not to be true, and pharmaceutical companies had historical profits from this health emergency crisis (10). For example, Pfizer moved from position 54 to 39 among transnational companies, becoming the number one in the ranking of transnational pharmaceutical companies, raising its profit from US$ 11.2 to 29.4 billion from 2019 to 2023 (11).

Patenting of health technologies plays a critical role in the monopoly power of companies, affecting the production, the supply decision-making and delaying the adoption of strategies to increase access with negative effects on people’s lives (10). Patent monopoly is one kind of capital accumulation strategy employed by pharmaceutical companies (12).

In this scenario, while some middle-income countries could develop COVID-19 technologies or engage in licensing and technology transfer and local production agreements, other LMICs could not procure any, bringing to the agenda the relevance of strengthening local manufacturing capacity in those countries (13). This included addressing intellectual property barriers, such as patents patents and trade secrets, which at the global level triggered a negotiation process, proposed by the governments of India and South Africa, to waive some articles of the agreement on Trade-Related Aspects of Intellectual Property Rights (TRIPS Agreement) (8).

There were also other approaches to address intellectual property barriers using voluntary mechanisms, such as the C-TAP initiative (14) and the Medicines Patent Pool (15), as well as bilateral licenses for technology transfer. TRIPS safeguards, such as compulsory licenses (16, 17) and patent oppositions (18–20) were also pursued in countries. Despite contributing to the COVID-19 response in some countries, this was not enough to overcome global inequity and timely meet the needs of the majority of LMIC. Patenting and other strategies (21) such as setting high prices even though the cost of production was low (22), continued as previously seen in other disease areas.

Evergreening is a range of practices to extend monopoly protection on existing products. The most common approach is filing several patent applications related to the same active pharmaceutical ingredient (API), covering not only the base compound (primary patent) but also salts, polymorphs, medical uses, combinations, formulations, dosage regimens, processes, etc. (secondary patents) (23). From a public health perspective, the multiple patent applications have several negative effects on access to technologies, such as the creation of a monopoly situation or its extension, bringing high prices and legal uncertainty for procurers and manufacturers.

This study aimed to analyze the evergreening approach of multiple patenting regarding technologies for SARS-CoV-2 and draw reflections on the consequences of this practice in the research agenda and access to health technologies in LMIC.

## 2 Methods

### 2.1 Selection of health technologies

There was an intentional selection of COVID-19 technologies as follows: three antivirals (favipiravir, remdesivir, and molnupiravir), a monoclonal antibody (sarilumab), and two non-conventional vaccine platforms (mRNA with lipid nanoparticle (LNP)—Moderna mRNA-1273—and viral vector—Oxford-AstraZeneca AZD1222). This selection was not based on the current best option for treatment and prevention (24).

### 2.2 Building of the patent landscape

The patent landscape for the selected technologies was built from existing landscapes publicly available, such as VaxPaL (25) and MedsPaL (26) databases, and complemented with a search at the commercial CAS Scientific Patent Explorer database (27), considering filing publicly available up to May 2023. The scope of the study was limited to patent applications filed through the Patent Cooperation Treaty (PCT) system and did not focus on regional or national patent applications and related status.

### 2.3 Classification of patent applications and analysis

The patent applications were classified according to the type of claims, following the UNDP guidelines for small molecules (28). These categories include Markush claims, selected compounds, polymorphs, enantiomers, salts, ethers, esters, compositions, doses, combinations, prodrugs, metabolites, processes, uses, and method of treatment. The same classification was adapted for the monoclonal antibody and for the vaccines.

To demonstrate the evergreening approach for each technology, a 20-year patent term was estimated for each patent application, starting from the international filing date at the PCT system, for the hypothetical scenario that they were filed and granted at the national level. All figures were created with BioRender.com.

## 3 Results

### 3.1 Antivirals

**Figure 1:**
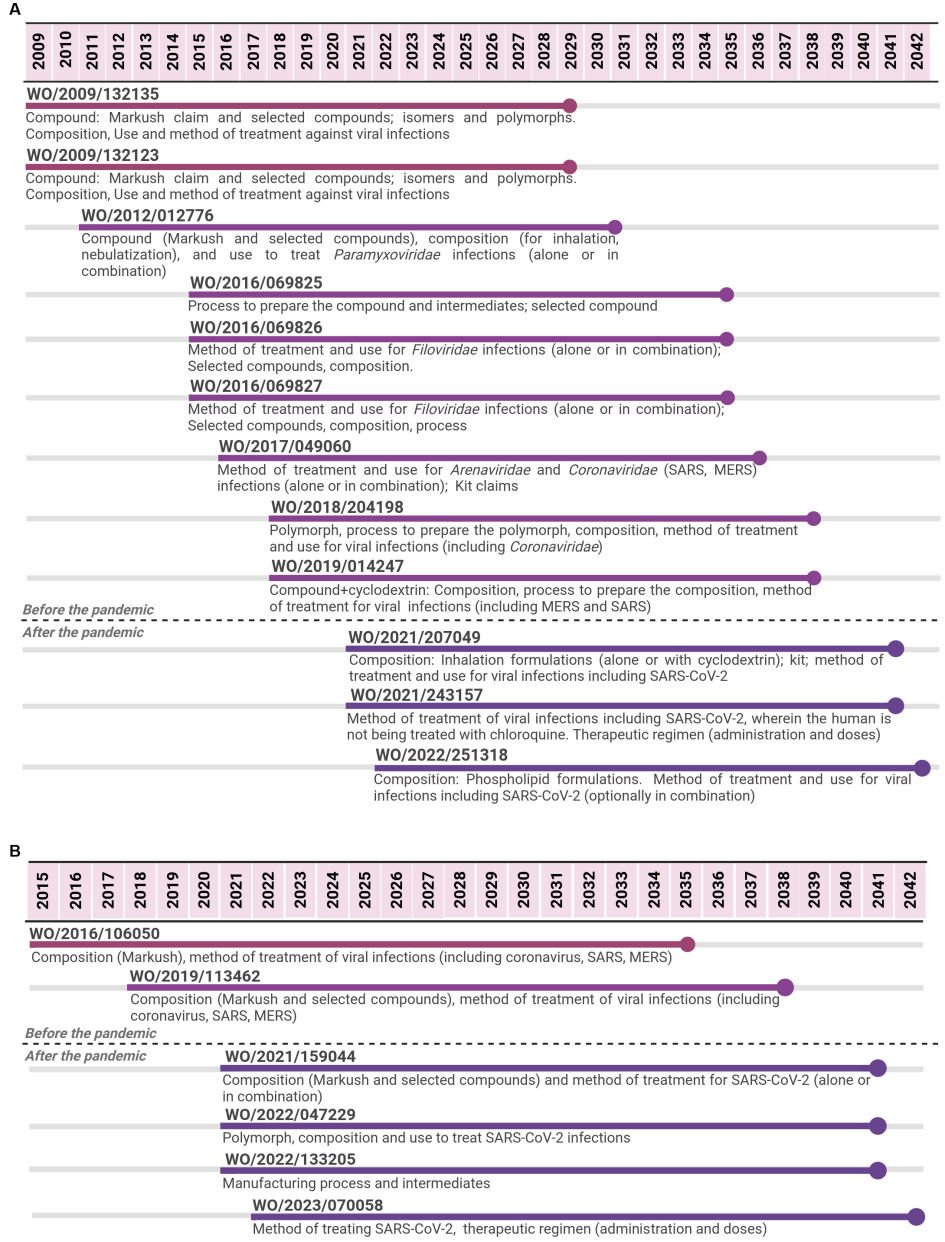
Timeline of potential patent protection related to remdesivir **(A)** and to molnupiravir **(B)**. The selection was based on international patent applications filed under the PCT system and estimates built on a 20-year patent term. The international patent applications are aligned according to the international filing date.

**Figure 2:**
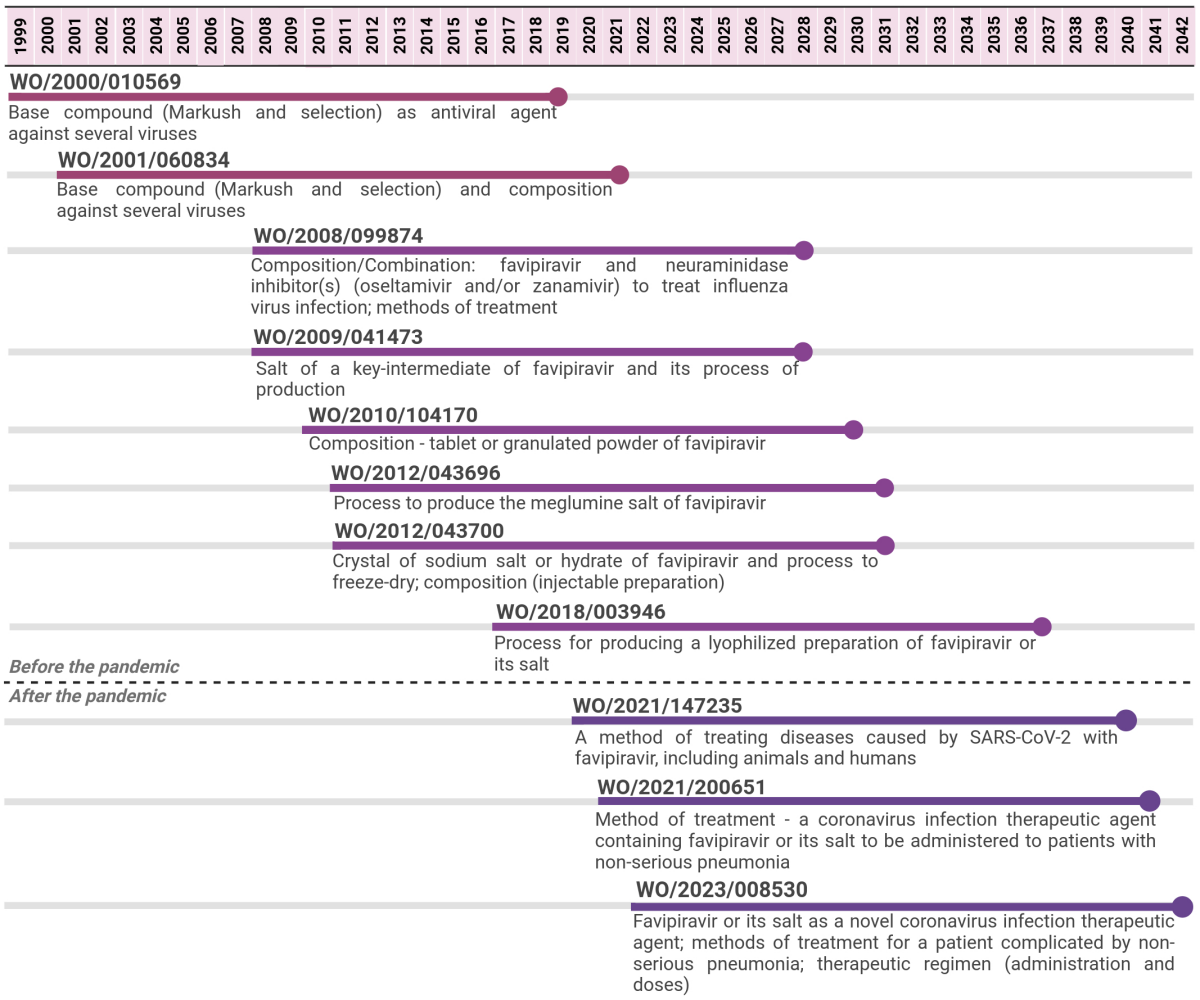
Timeline of potential patent protection related to favipiravir. The selection was based on international patent applications filed under the PCT system and estimates built on a 20-year patent term. The international patent applications are aligned according to the international filing date.

The three antivirals assessed with regard to patenting are represented in Figures 1, 2.

For remdesivir, 12 PCT applications filed by Gilead were identified from 2009 to 2022. The last application is expected to expire in 2042, 13 years after the expiring date of the first application, implying a total of 33 years of monopoly. The first two applications focused on the API covered by Markush claims and selected compounds, including isomers and polymorphs. Markush type of claims refer to a chemical structure with specific parts allowing multiple substitutions that result in several compounds (28). Initial PCT applications also covered aspects related to the composition (formulation and dosage form) and the medical indication (methods of treatment or use claims). With regard to medical indication, initial claims were aimed at viral infections as a broad term to cover many options. Subsequent PCT applications continued the trend of focusing on compositions and method of treatment/use, narrowing the medical indication to specific families of viruses (*Paramyxoviridae* or *Filoviridae*); as well as on processes to produce the API. From 2017 to 2019, PCT applications included the *Coronaviridae* family as a medical indication, mentioning MERS, SARS, polymorph, and combination claims. PCT applications filed after the pandemic narrowed even more to focus on compositions and methods of treatment/use for SARS-CoV-2.

For favipiravir, 11 PCT applications filed by Toyama Chemical were identified from 1999 to 2022. The last application is expected to expire in 2042, 23 years after the expiring date of the first application, implying 43 years of monopoly. The first two applications focused on the API covered by Markush claims and selected compounds as broad antiviral agents. Subsequent PCT applications, from 2008 to 2017, focused on other aspects of the API, such as its salt forms or process to produce them, pharmaceutical compositions, and combinations of the API with other antivirals for use in influenza. The three PCT applications filed after the pandemic narrowed the focus to SARS-CoV-2 and several forms of COVID-19 disease (method of treatment and use claims).

For molnupiravir, six PCT applications filed by Emory University were identified from 2015 to 2022. The last application is expected to expire in 2042, 7 years after the expiring date of the first one, implying 27 years of monopoly. Most PCT applications were filed in a short period of time since the beginning of the pandemic. The first two applications cover the API (Markush and selected compounds) through composition claims and the medical indication as broad antiviral agents. Among the different types of viruses mentioned, coronavirus, SARS, and MERS were included. The subsequent four PCT applications, filed after the pandemic, narrowed the scope of protection to specific compounds through composition claims, a medical indication to SARS-CoV-2 through the method of treatment and use claims, API manufacturing process, and key-intermediates, as well as polymorphs.

### 3.2 Monoclonal antibody

**Figure 3:**
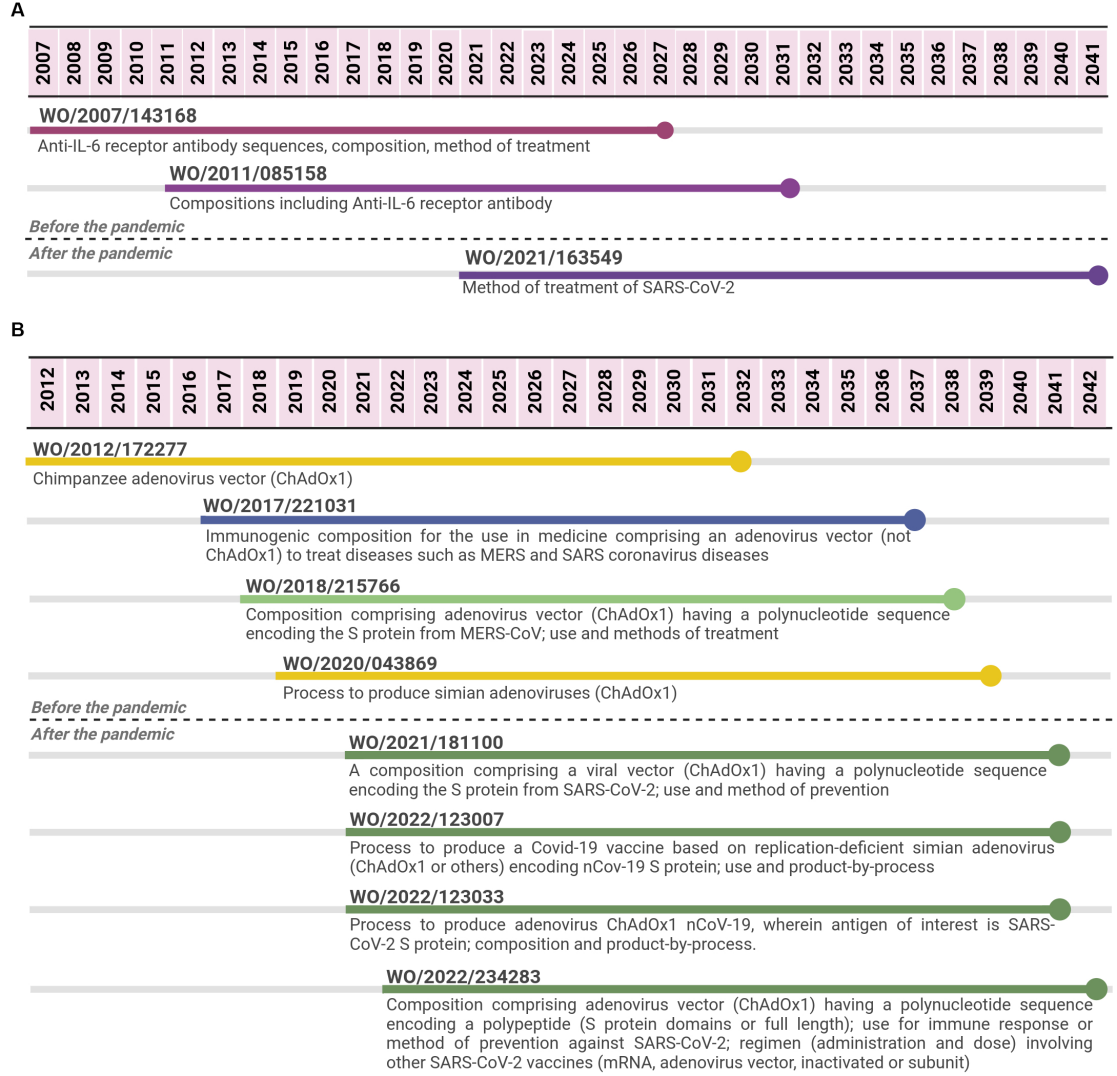
Timeline of potential patent protection related to sarilumab **(A)** and to Oxford-AstraZeneca viral vector vaccine **(B)**. The selection was based on international patent applications filed under the PCT system and estimates built on a 20-year patent term. The international patent applications are aligned according to the international filing date. Reference for Oxford vaccine color code **(used in B)**: related to the adenovirus vector ChAdOx1 (yellow); related to the viral vector platform applied for coronaviruses diseases (blue); related to adenovirus vector (ChAdOx1) encoding spike protein from MERS-CoV (light green); related to adenovirus vector (ChAdOx1) encoding spike protein from SARS-CoV-2 (first-generation vaccine) (dark green).

The sarilumab case was selected as an example of a biological therapeutic. Three PCT applications filed by Regeneron Pharmaceuticals were identified from 2007 to 2022. The last PCT application was co-filed by Sanofi Biotechnology and is expected to expire in 2041, 14 years after the expiring date of the first application, implying 34 years of monopoly. The first PCT application focused on the API (specific monoclonal antibody), compositions and medical indication (method of treatment and use claims) for an IL-6-mediated disease or disorder, e.g., arthritis. Meanwhile, the second one focused on aspects of the formulation (excipients and concentration) involving the API. The third PCT application was filed after the pandemic and narrowed the medical indication for SARS-CoV-2 (method of treatment or use claims) (Figure 3).

### 3.3 Vaccines

For the viral vector case, 8 PCT applications were identified from 2012 to 2022 (Figure 3B), 4 applications were filed within a period of 7 years (between 2012 and 2019), and 4 additional applications were filed within 2 years (2021–2022). The four initial PCT applications were filed by Isis Innovation or Oxford University Innovation (OUI), which are related to the patent management at Oxford University (29). After the pandemic, two PCT applications were filed by OUI, one by AstraZeneca as the only applicant and the last one by both AstraZeneca and OUI as co-applicants. The four PCT applications filed before the pandemic either focused on the chimpanzee adenovirus vector (ChAdOx1) and its process of production or on compositions comprising an adenovirus vector (ChAdOx1 or others) with a sequence encoding an antigen for the medical application in MERS and/or SARS coronaviruses. One case specified that the antigen was MERS-CoV spike protein. The four PCT applications filed after the pandemic focused on SARS-CoV-2. Two applications were related to compositions comprising a viral vector, including adenovirus vector ChAdOx1, a polynucleotide sequence encoding SARS-CoV-2 spike protein. They had claims related to its application as vaccines (methods of prevention or use claims), including administration schedule, dosing, and mixing different vaccine options. The other two PCT applications were mainly focused on the process of producing COVID-19 vaccines based on simian adenovirus, including ChAdOx1.

**Figure 4:**
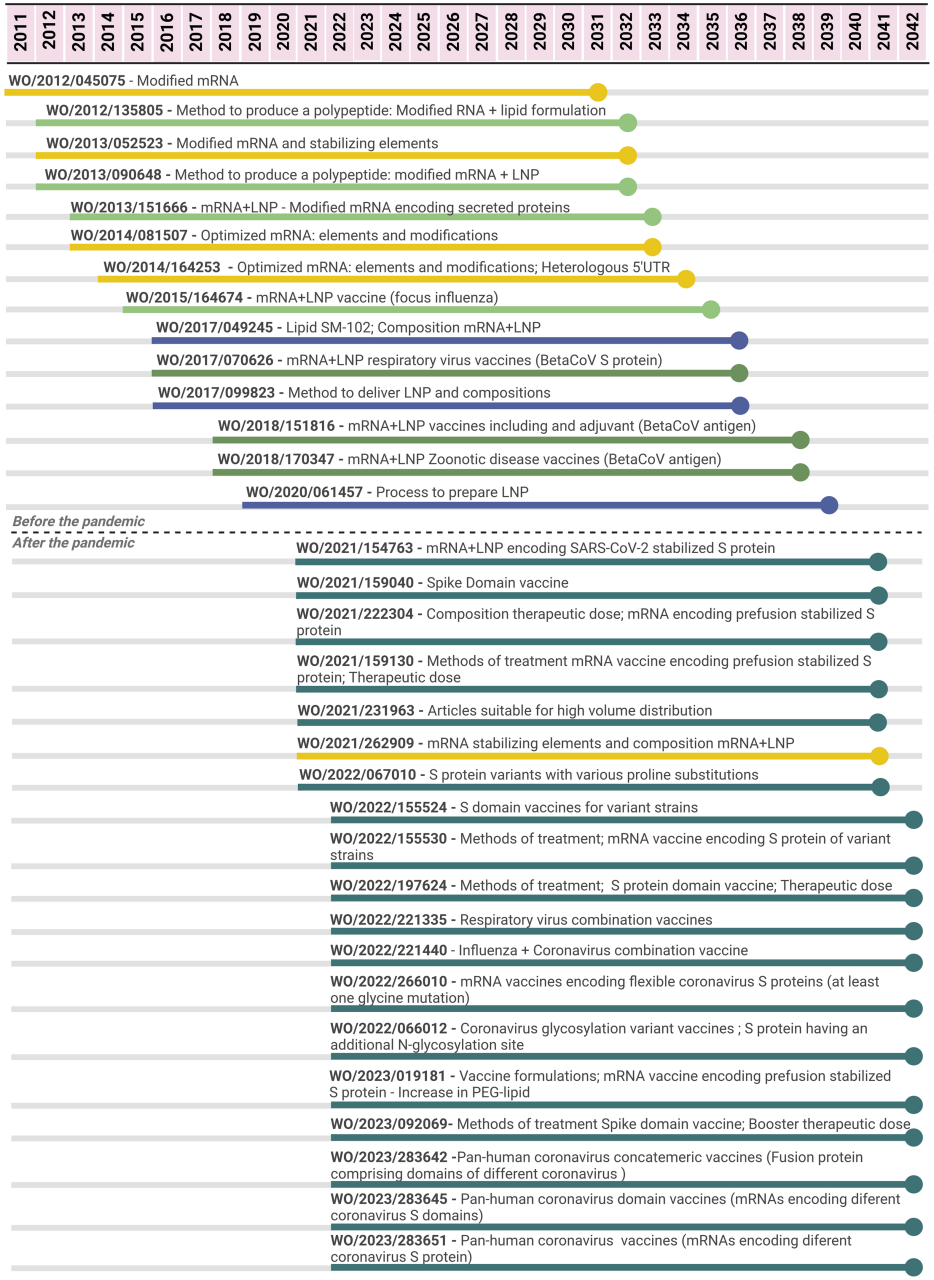
Timeline of potential patent protection related to mRNA-1273 vaccine developed by Moderna. The selection was based on international patent applications filed under the PCT system and estimates built on a 20-year patent term. The international patent applications are aligned according to the international filing date. Color code: related to the mRNA elements and modifications (yellow); related to delivery compounds and compositions (blue); related to the mRNA + LNP platform (light green); related to the mRNA + LNP platform applied for coronavirus diseases (medium green); related to the mRNA + LNP platform applied for SARS-CoV-2 (dark green).

In relation to the mRNA-1273 vaccine, 33 PCT applications filed by Moderna were related to the vaccines from 2011 to 2022 (Figure 4); 14 applications were filed between 2011 and 2020, while 19 were filed after the pandemic (period of 2.5 years). The initial seven PCT applications did not specifically cover vaccines or compositions comprising viral antigens; claims were more general with regard to the parts of the technology platform. For example, four of them focused on the RNA nucleoside modifications, stabilizing elements of the molecule, and methods to produce a polypeptide or to increase their levels in a cell, tissue, or organism. Three other applications refer to mRNA formulated in LNP (or a lipid formulation and methods of production of a polynucleotide in a cell, tissue, or organism). Between 2015 and 2020, there were PCT applications focusing on the platform mRNA + LNP as vaccines, including claims with a broad scope of possible antigens, such as “a betacoronavirus antigen.” These applications include mainly claims on compositions (vaccine) and methods of prevention, covering different aspects of the vaccine. There were claims referring to Markush formulas for the individual lipids. Three patent applications focused on LNP for the delivery of nucleic acids with claims related to individual lipids (compound claims), processes to produce LNP, and methods of treatment/use. One of them is the first one referring to the specific ionizable lipid SM-102, included in the Moderna mRNA-1273 vaccine (30), and as LNP composition (including the other lipids of the vaccine).

As observed in the molnupiravir case (Figure 1), most PCT applications for the mRNA-1273 vaccine were filed in a short period of time since the beginning of the pandemic. In relation to those PCT applications filed after the pandemic, 18 of 19 are specific for SARS-CoV-2. The first application covered all the components of the mRNA-1273 vaccine, including the mRNA molecule encoding “SARS-CoV-2 Spike protein having a double proline stabilizing mutation” (or a fragment), the composition involving mRNA+LNP with specific lipids composing the LNP and methods of prevention. Subsequent PCT applications provided a diversity of claims covering different aspects of mRNA+LNP vaccines for SARS-CoV-2, related to the components of the mRNA-1273 vaccine, but not limited to it. For example, there were PCT applications related to mRNA molecules encoding different domains of the spike protein (“domain” vaccines); encoding for a Spike protein of a variant circulating SARS-CoV-2 virus strain (full length or domain vaccine); a vaccine having 2–15 mRNA molecules encoding for antigens of different respiratory viruses (combination); encoding Spike proteins with particular mutations or from different coronaviruses for a pan-human coronavirus vaccine. These PCT applications referred to both aspects of the vaccine covering mRNA coding sequences and stabilizing elements as well as LNP composition, including the protection of the mRNA molecule, composition, and method of treatment/use. Several PCT applications focused on “methods of prevention,” including administration schedule, dosing, and combinations.

## 4 Discussion

In this study, we were able to analyze some of the evergreening strategies used by different applicants, academic institutions or pharmaceutical companies, for either creating or extending the protection of different pharmaceutical technologies beyond 20 years or establishing a complex net of applications covering, e.g., a range of possibilities of mRNA+LNP vaccine developments. The cases have shown that companies and academic institutions pursued patent filing activity on specific therapeutic and vaccines, and in some cases, this activity was more intense after the pandemic.

For the antivirals, the common trend in the patenting approach was that initial patent applications comprised broad claims referring to both compounds (Markush claims) and medical indications (as antiviral agents). Then, the focus narrowed to specific compounds (selected compounds) and specific medical indications (SARS-CoV-2).

These initial patent filings targeting a broad spectrum of viruses’ families are associated with the R&D performed prior to the COVID-19 pandemic. Remdesivir and favipiravir are RNA-dependent RNA polymerase (RdRp) inhibitors that have been explored up to clinical studies. Remdesivir was initially tested against filoviruses causing Ebola and Marburg diseases, reaching the clinical trial phase (but not getting the US FDA approval), and later showing activity in animal models against both SARS-CoV-1 and MERS-CoV (31, 32). Favipiravir got market approval against influenza in Japan in 2014 (33).

Molnupiravir has shown activity against diarrhea virus, hepatitis C virus, norovirus, chikungunya virus, Ebola virus, influenza viruses, syncytial viruses, CoV, Venezuelan equine encephalitis virus, and coronaviruses including SARS, MERS and SARS-CoV-2 (34), reflected in the patent filings identified, but it only reached the clinical trial phase with SARS-CoV-2. For sarilumab, although there was fewer patent filing activity (only three applications), PCT applications were related to rheumatoid arthritis, its initial medical indication approved (35), followed by the method of treatment on SARS-CoV-2.

There was renewed attention to repurposing drugs during the COVID-19 pandemic. Drug repurposing can be defined as “researching new indications for already approved drugs or advancing previously studied but unapproved drugs” (p.1) (36). Remdesivir, favipiravir and sarilumab are therapeutics resulting from this approach. It has been adopted based on the assumption, among others, that the development process can be shortened, cheaper, and with lower risk in comparison with traditional approaches. However, pharmaceutical companies are also known for de-prioritizing or abandoning promising drug candidates during the R&D process, and intellectual property was identified as the second main barrier to drug repurposing (36). For example, intense patent filings on a compound prevent other institutions from exploring R&D activities on such compounds, unless they get a license with the patent holder, which can be time-consuming and difficult to negotiate. It is common practice for drug developers to patent a variety of compounds during a drug development project, protecting not only the final candidate but many if not all of the “semi-finalist” compounds (37). Therefore, this approach allows companies to protect their shelved compounds and prevent competitors from working on similar promising drug candidates (36). This is reflected in the three antivirals analyzed, wherein initial patent applications focused on Markush type of claims and subsequent applications targeted specific compounds. Markush claims in patent applications bring complex issues because one patent application can block the R&D, production, and commercialization of thousands of molecules. Some studies showed a growing use of Markush claims in developing countries (28).

Even for a new chemical entity for COVID-19, such as nirmatrelvir, the originator company as a sole supplier in the market refused to allow the combination with other compounds in clinical trial studies in LMIC, showing the monopoly power during efforts to implement R&D to meet a public health need (38).

From an access perspective, the patent protection of essential medicines has created a monopoly situation that allows companies to charge high prices, as seen over the past nearly three decades for the antiretrovirals for HIV infection and lately for direct acting-antivirals for hepatitis C (39, 40) and other disease areas. The evergreening strategy worsens the patent-related access challenges by not only extending the term of the market monopoly situation, but also increasing legal uncertainty for procurement processes (41), due to the different status of those applications and the chilling effect (of even non-blocking secondary patents), preventing efforts to import or engage in local production for affordable alternatives (generics or biosimilars).

The COVID-19 pandemic has brought several lessons regarding the effects of monopoly on access inequity to health technologies (8). One lesson from the COVID-19 pandemic is that the monopoly situation not only affects prices but also the supply in a health emergency (9). In 2020, after being the first therapeutic to get emergency use authorization by the US FDA, almost all of the world’s supply of remdesivir was procured by the US government, which shows that HIC prioritized their national interests (42, 43). Additionally, the country paid US$ 2,340 per 5-day treatment, while the estimated cost of manufacturing was US$ 0.93 per day (22, 44).

Depending on the approach taken on patent examination, the filing of secondary patent applications for an old compound that gets market approval might represent the possibility of getting a monopoly over a newly approved drug. Therefore, those applications will be the actual monopoly in the country when a compound reaches the market. Patent applications related to the salt form of tenofovir’s prodrug (tenofovir disoproxil fumarate) was the main approach for the US company to pursue patent monopoly in 2000 as the compound was disclosed since the 1980s by researchers in former Czechoslovakia (45, 46).

The second lesson from the COVID-19 pandemic is a new challenge to the way the current intellectual property system operates: the time period from when a patent application is first filed in the country of origin, published at the international level through the PCT system, until it gets national level might take up to 30 months from the priority date. As some technologies were developed and got market authorization for COVID-19 in a short period of time, from the time while the international patent application was not published, there was a gap of information about the full picture of the patent landscape and whether a patent application would enter in the national phase in a country or not. For example, while remdesivir got the Emergency Use Authorization in the United States in May 2020 and later the approval in October of the same year (47), the publication of the first PCT application involving the method of treatment for SARS-CoV-2 was only nearly 1 year later, on October 2021. PCT applications covering all the aspects of the COVID-19 mRNA vaccines were only made public in 2021, while the vaccines were approved in some countries at the end of 2020. As the present analysis shows, the number of patent applications increased after the pandemic period. Therefore, the full picture of the patent landscape not being entirely and timely public in a country may limit the space for and delay the use of TRIPS public health safeguards to promote access.

From a public health perspective, the TRIPS agreement allows countries to exclude from patentability “diagnostic, therapeutic and surgical methods for the treatment of humans or animals” (48), which, if adopted in the national legislation, would allow the rejection of several patent applications and claims related to new medical indications for those compounds and, therefore, prevent the negative effects of evergreening.

Examples such as favipiravir and sarilumab, which got market approval for a prior medical indication, also fall in the category of “second medical use,” which accounts for multiple patent applications in the pharmaceutical field. A public health perspective applied to those cases would reject those types of patent applications on the following grounds: it is a discovery of a property; it lacks technical character, therefore, it is not an invention; it lacks novelty, given the compound and its process of production are known; it lacks industrial application because the effects happen in the body (28).

For the two vaccine platforms analyzed, there was intense patent filing, which was intensified after the pandemic. Regarding antivirals, evergreening reflects multiple patents involving the API itself (compound, salts, polymorphs, hydrates, and process of production), including the API (pharmaceutical formulation and dosage forms) or applying the API for certain indication (methods of treatment and use) (41). However, evergreening for viral vector and RNA-based vaccines could be differently analyzed: while there are applications related to the technology platform itself or its components, those related to ‘medical indication for certain disease’ will be related to which antigen(s) of specific pathogen to produce an immune response is encoded by the genetic material sequence; which is considered the active substance by the regulatory authorities (49).

For the viral vector case, two PCT applications filed before the pandemic focused on the chimpanzee adenovirus vector (ChAdOx1) and its process of production, while two others anticipated its applications to the existing coronaviruses at that time and the potential of Spike protein as a target vaccine candidate (composition and method of treatment/use claims). After the pandemic, the four PCT applications focused on spike protein from SARS-CoV-2 and viral vector ChAdOx1, including processes of production and variations in the sequence related to spike protein.

In the mRNA vaccine case, initial PCT applications focused on either mRNA modifications or optimization along with LNP as delivery systems. With regard to LNP, several claims focused on specific lipids composing the LNP, including the SM-102, and their proportions, as well as the process of production or synthesis. mRNA+LNP vaccine platforms encoding betacoronavirus antigens, including Spike protein, were also targets prior to the pandemic (Figure 4).

After the pandemic, a common aspect among vaccine PCT applications was the focus on SARS-CoV-2 or broadly coronavirus vaccines. Although similar trends can relate to those for small molecules, such as intense patenting with methods of treatment or prevention, the high number of claims may relate not only to components of the first-generation approved vaccine but also to potentially future ones. These applications include broad claims referring to variants or fragments of a certain sequence and even to a general “SARS-CoV-2 antigen” resulting in a sort of repetition on the scope of protection across different patent applications and, at the same time, multiple vaccine candidates’ coverage.

Previous analysis has shown that in the mRNA patenting space, multiple players were filing claims related to the same component of either the mRNA or LNP indicating an overlap in the patent filings. For example, in PCT applications from Moderna, BioNTech, and CureVac, there were similar claims on mRNA modification (with methyl pseudouridine) and components of LNP (cholesterol and DSPC). This approach poses legal risks to companies with technologies already approved as well as those in the R&D pipeline. Broad claims, if granted in a certain jurisdiction, may encompass knowledge of the state of the art essential to the platform development (21).

The approach to pursuing broad claims in the vaccine field has already been described. Importantly, patenting on vaccines usually involves the strategy of adopting “broad, non-specific claim language” and “overly general language in patent claims concerning the scope of the inventions” (50). From a commercial perspective, pursuing broad claims in the mRNA space is an approach to include and anticipate competitors’ activities, which may result in “maximizing the leverage of each patent estate, which will be useful in enforcement activity, licensing deals, and set-up for exit through merger or acquisition” (51). However, from a public health and development perspective, this can become a disincentive for manufacturers to engage in the development process of affordable versions, suitable to LMIC public health needs.

The third lesson from the COVID-19 pandemic is that expanding manufacturing capacity for regional supply is critical to prepare for future pandemics and there are several challenges in doing so, including IP barriers, such as patents and trade secrets (8, 9, 52). The increased patent filing related to vaccines adds a key layer of complexity and legal uncertainty for governments and manufacturers in LMIC to pursue efforts of local production.

This study explored the evergreening approach to selected COVID-19 technologies and analyzed the content of multiple patent applications related to those technologies. The number of patent filings during the pandemic, if filed at the national level, confirms the challenges posed to countries in addressing access, due to the monopoly situation (*de facto* or de jure) over those technologies, including legal uncertainty to governments and producers in LMIC. In addition to the different measures to protect public health pursued over the past years, efforts to adopt a public health approach in patent examination should be considered to prevent the granting of undeserved patents.

Life-saving technologies can only change the course of a pandemic if equitable access is guaranteed to all those who need it worldwide.

## Data availability statement

The original contributions presented in the study are included in the article/supplementary material, further inquiries can be directed to the corresponding author.

## Author contributions

MB: Conceptualization, Data curation, Formal analysis, Investigation, Methodology, Writing – original draft, Writing – review & editing. MP: Conceptualization, Data curation, Formal analysis, Methodology, Writing – original draft, Writing – review & editing. CS: Conceptualization, Data curation, Formal analysis, Methodology, Writing – original draft, Writing – review & editing. SK: Conceptualization, Formal analysis, Funding acquisition, Project administration, Writing – review & editing. OM: Conceptualization, Formal analysis, Funding acquisition, Writing – review & editing. GC: Conceptualization, Data curation, Formal analysis, Investigation, Methodology, Supervision, Writing – original draft, Writing – review & editing.
